# The Microbial Signature Provides Insight into the Mechanistic Basis of Coral Success across Reef Habitats

**DOI:** 10.1128/mBio.00560-16

**Published:** 2016-07-26

**Authors:** Alejandra Hernandez-Agreda, William Leggat, Pim Bongaerts, Tracy D. Ainsworth

**Affiliations:** a Australian Research Council Centre of Excellence for Coral Reef Studies, James Cook University, Townsville, Australia; b The College of Public Health, Medical and Veterinary Sciences, James Cook University, Townsville, Australia; c Australian Research Council Centre of Excellence for Coral Reef Studies, University of Queensland, Brisbane, Australia

## Abstract

For ecosystems vulnerable to environmental change, understanding the spatiotemporal stability of functionally crucial symbioses is fundamental to determining the mechanisms by which these ecosystems may persist. The coral *Pachyseris speciosa* is a successful environmental generalist that succeeds in diverse reef habitats. The generalist nature of this coral suggests it may have the capacity to form functionally significant microbial partnerships to facilitate access to a range of nutritional sources within different habitats. Here, we propose that coral is a metaorganism hosting three functionally distinct microbial interactions: a ubiquitous core microbiome of very few symbiotic host-selected bacteria, a microbiome of spatially and/or regionally explicit core microbes filling functional niches (<100 phylotypes), and a highly variable bacterial community that is responsive to biotic and abiotic processes across spatial and temporal scales (>100,000 phylotypes). We find that this coral hosts upwards of 170,000 distinct phylotypes and provide evidence for the persistence of a select group of bacteria in corals across environmental habitats of the Great Barrier Reef and Coral Sea. We further show that a higher number of bacteria are consistently associated with corals on mesophotic reefs than on shallow reefs. An increase in microbial diversity with depth suggests reliance by this coral on bacteria for nutrient acquisition on reefs exposed to nutrient upwelling. Understanding the complex microbial communities of host organisms across broad biotic and abiotic environments as functionally distinct microbiomes can provide insight into those interactions that are ubiquitous niche symbioses and those that provide competitive advantage within the hosts’ environment.

## IMPORTANCE

Corals have been proposed as the most diverse microbial biosphere. The high variability of microbial communities has hampered the identification of bacteria playing key functional roles that contribute to coral survival. Exploring the bacterial community in a coral with a broad environmental distribution, we found a group of bacteria present across all environments and a higher number of bacteria consistently associated with mesophotic corals (60 to 80 m). These results provide evidence of consistent and ubiquitous coral-bacterial partnerships and support the consideration of corals as metaorganisms hosting three functionally distinct microbiomes: a ubiquitous core microbiome, a microbiome filling functional niches, and a highly variable bacterial community.

## INTRODUCTION

Identifying specific bacteria that provide critical functional contributions to a host organism (and the ecosystem it is part of) requires an understanding not only of the bacterial population, but of the persistence and stability in time and space of both the microbial functional niches and the bacteria that utilize them. This is a challenging task given that bacterial communities tend to be both highly diverse and highly variable, and functional niches can be extremely difficult to identify in highly complex communities. Differentiating the bacterial associations with corals is an example of this challenge. As in all other natural systems, bacterial communities associated with corals are proposed to have important functional contributions to their health (1, 2), nutrition (3, 4), and nutrient cycling (5, 6). However, the microbiome associated with corals is one of the most complex and diverse studied to date (7). Corals harbor thousands of bacterial phylotypes, and the communities they form vary structurally (composition and abundance) between coral species across geographical, spatial, and temporal scales (8–10).

The structure of bacterial communities in corals has been shown to be highly variable and to respond to many biotic and abiotic factors (8–10). Biological events, such as algal competition, reproduction, and diseases, as well as changes in environmental variables, including temperature, pH, nutrients, and dissolved organic carbon, generate shifts in the composition, richness, and abundance of coral-associated bacteria (11–16). Moreover, the responses of the bacterial community (and community members) differ between host coral species, as well as between stimuli (17). Thus, while there is evidence that the coral-associated bacterial communities change in response to disturbance, there is substantial confusion with regard to the impact of underlying natural variability in patterns of coral-associated bacteria. For example, the coral endosymbiosis with the dinoflagellate *Symbiodinium* generates patchy microhabitats with different environmental conditions within an individual host (18). Bacterial communities differ along the host colony and between niche compartments, such as the surface mucus, the symbiosome, and the skeleton (19, 20). Therefore, despite over a decade of research documenting coral-associated bacteria, the identities of specific bacteria playing important roles in corals and their responses to biotic and abiotic variables remain poorly characterized. A core microbiome approach, focused on the identification of ubiquitous bacteria rather than highly abundant bacteria, has been suggested as an alternative for differentiating stable and functionally significant coral-bacterial interactions, overcoming the complexity of bacterial communities, and functionally differentiating bacterial symbioses (19, 21).

**FIG 1:**
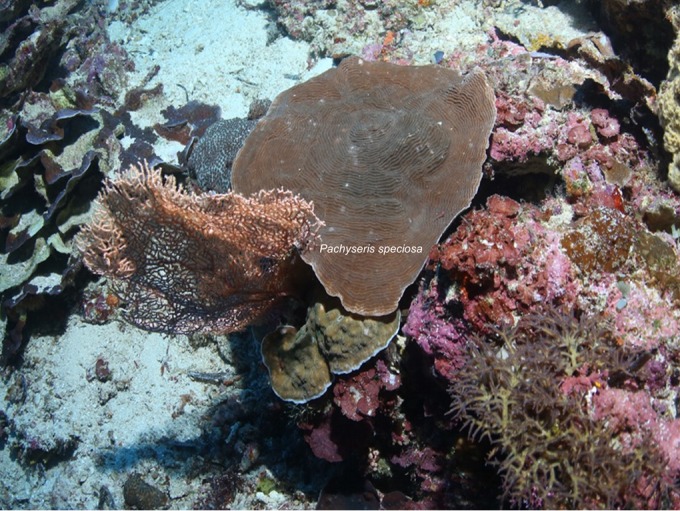
Host *Pachyseris speciosa*, a depth-generalist coral.

The high degree of variability of the bacterial communities, the complexity of the coral host habitat and the coral reef environment, and the difficulties in identification of functionally important bacteria in corals have together contributed to substantial uncertainty in regards to the identities, roles, and significance of bacterial symbioses on corals. Addressing this uncertainty requires comprehensive analysis of the diversity, commonality, and rarity of bacterial phylotypes on coral hosts. To do so, sample sizes (number of individual hosts investigated) need to be greatly increased, as does the diversity of reef habitats sampled for the same host species. The environmental-generalist coral *Pachyseris speciosa* (Fig. 1) is one coral species that is found in most reef environments of the Great Barrier Reef (GBR) and the Coral Sea (22, 23), and as such, it represents an ideal model to test the bacterial-persistence hypothesis (i.e., the presence of ubiquitous bacteria within hosts across diverse habitats). The Great Barrier Reef represents the largest coral reef ecosystem in the world, extending over 2,300 km (14° of latitude) and encompassing a surface area of 348,000 km^2^. The adjacent Coral Sea Commonwealth Marine Reserve (CSCMR) is located east of the GBR and represents a large region (989,842 km^2^) containing numerous coral atolls that, to date, have remained largely unstudied (Coral Sea Commonwealth Marine Reserve—Overview, http://www.environment.gov.au/topics/marine/marine-reserves/coral-sea/overview; accessed 9 February 2016). Host-microbiome interactions and/or symbioses are potential mechanisms by which environmental-generalist coral species are able to successfully occupy a broad range of reef habitats. Here, we characterize the bacterial communities of *P. speciosa* samples from reefs across the GBR and the Coral Sea (CS).

## RESULTS AND DISCUSSION

We propose that the coral holobiont of the environmental-generalist coral *P. speciosa* should be considered as three functionally different fractions, as follows: first, a ubiquitous core microbiome consisting of a small group of bacteria that are persistent across spatial scales and along depth gradients and are likely to be symbiotic; second, a spatially and/or regionally explicit core microbiome that is composed of bacteria found consistently in individuals within specific environmental regimes and that likely aid coral success within the environment; and third, a highly variable bacterial community that is responsive to processes occurring at both large (hundreds of kilometers; for example, reef regions) and small (meters; for example, depth ranges) spatial scales.

### The bacterial community of *P. speciosa.*

An operational taxonomic unit (OTU) database containing 4,176,251 high-quality reads and comprising 173,690 OTUs was generated for all corals (*n* = 123) sampled within the study. OTUs with a percentage of occurrence of <5% were excluded (i.e., those found in less than 6 of the 123 coral samples), as these were considered to be transient members, which reduced the number of OTUs to 4,446 phylotypes.

**FIG 2:**
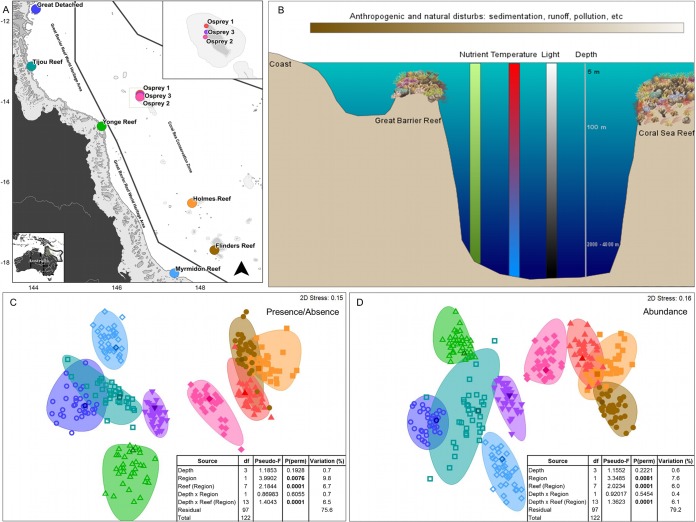
Biographical differences in coral-associated bacterial (holobiont) structures of *P. speciosa*. (A) Study sites in the Great Barrier Reef (GBR, left) and Coral Sea (CS, right). (B) Environmental factors changing with depth and with closer proximity to the coast. (C) Differences in bacterial composition between GBR and CS and between reefs (nMDS, Bray-Curtis; inset, PERMANOVA table). (D) Differences in bacterial abundance between GBR and CS and between reefs (nMDS, Bray-Curtis, data: fourth root transformed, sample standardized by total; inset, PERMANOVA table). Factors are as follows. (i) Region: Great Barrier Reef (GBR) and Coral Sea (CS). (ii) Reef (Region): Great Detached (GBR), Tijou Reef (GBR), Yonge Reef (GBR), Myrmidon Reef (GBR), Osprey 1 (CS), Osprey 2 (CS), Osprey 3 (CS), Holmes Reef (CS), and Flinders Reef (CS). (iii) Depth: 10 m, 20 m, 40 m, and 60 to 80 m. Variation (%) refers to components of variation.

We find that the bacterial community structures are different between regions, reefs, and in some reef locations, between depths (Fig. 2A, C, and D; see also Tables S1 to S6 in the supplemental material). Biotic and abiotic processes occurring at those regional scales (factor Regions [see Materials and Methods], scale of 10 to 100 km) are likely to substantially influence coral holobiont bacterial communities, both in terms of composition and abundance. The Coral Sea reefs are in oceanic waters, where variables like flow rate, mixing and tidal currents, temperature, and concentration of nutrients are vastly different than in the reefs of the Great Barrier Reef lagoon (Fig. 2B). Bacterioplankton, biofilms, and coral holobiont bacterial communities have previously been shown to be responsive to alterations in water quality (16, 24, 25). Moreover, environmental variables like nutrient concentration, temperature, and light vary significantly across a reef depth gradient (Fig. 2B). Differences in water quality and oceanography between the reefs from the GBR and the CS could be affecting the structure of coral-associated bacteria. However, there is currently a substantial lack of information about how bacterial communities in corals change in relation to reef depth, since (i) variations in these factors are site specific (evidenced in our results), (ii) these factors have been evaluated in isolation, and (iii) bacterial communities in corals have been studied principally at shallow depths (0 to 30 m) and only sparsely studied in mesophotic reef zones (depths from 30 m to 200 m) (4, 26, 27). Our findings here support previous studies that have indicated that coral-associated bacterial communities (holobionts) are highly responsive to environmental conditions that can change over distances ranging from meters (as in depth gradient) to hundreds of kilometers (between reefs and regions).

### Persistent bacteria: a core microbiome.

**FIG 3:**
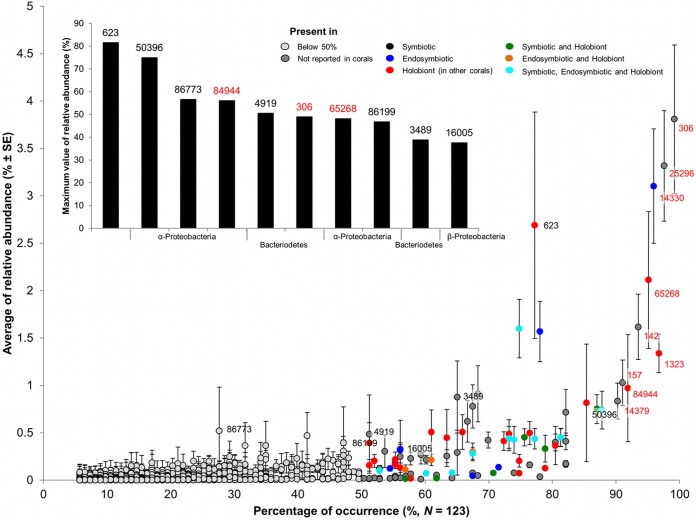
Comparison of average relative abundances and percentages of occurrence. Each point represents an operational taxonomic unit (OTU). Point colors indicate microhabitats where these OTUs have been reported previously (NCBI, identities of ≥97%). Labels display OTU numbers; the top nine OTUs with the highest percentages of occurrence are in red. See Table S7 in the supplemental material for complete taxonomic identification. Bar chart shows the top 10 OTUs with the highest maximum values of relative abundance and their taxonomic classification.

Our results provide the most comprehensive evidence to date for the presence of a small group of bacteria that are ubiquitously associated with corals regardless of abiotic environmental factors. Despite the high diversity and variability of coral-associated bacteria found across spatial scales, we found that of the 173,690 bacterial phylotypes recovered from *P. speciosa*, only 9 are present in over 90% of coral individuals (Fig. 3, red labels), and only 97 are found in over 50% of individual coral colonies. Traditionally, coral-associated bacteria have been analyzed by focusing on highly abundant bacteria, regardless of their occurrence across individual corals. However, abundance measures are known to be biased by the methods of sample preparation, sample handling, and data generation (28–30). Our results clearly show that studies focusing on the importance of highly abundant bacteria overlook the frequently occurring bacteria that are generally in relatively low abundance and/or rare in whole-colony (or holobiont) community analyses (Fig. 3, inset). Meta-analysis (Nucleotide BLAST of the National Center for Biotechnology [NCBI] database) reveals that of the 97 bacterial phylotypes consistently present in over 50% of samples, 49 have been previously reported in specific coral microhabitats (symbiont and endosymbiont) and/or as part of coral microbiota (see Table S8 in the supplemental material). These results highlight the importance of considering persistence instead of abundance to define potentially functionally important bacteria in association with reef-building corals. We also find that only two bacterial phylotypes are both highly persistent (ubiquitous) and highly abundant within community analyses (OTUs 306 and 25296) (Fig. 3). These two bacteria are both novel in reports on bacteria within the coral microbiome. The novel identification of two highly abundant and ubiquitous bacteria is likely the result of two factors: the large sampling design of the current study (*n* = 123 corals, the largest undertaken to date) and the application of Illumina sequencing technology, allowing great depth of sequencing within the coral microbiome. This result highlights that the application of sampling designs with greater depth of coverage is likely to be crucial in the identification of potentially symbiotic bacteria within high-diversity (>170,000) coral community analyses.

**FIG 4:**
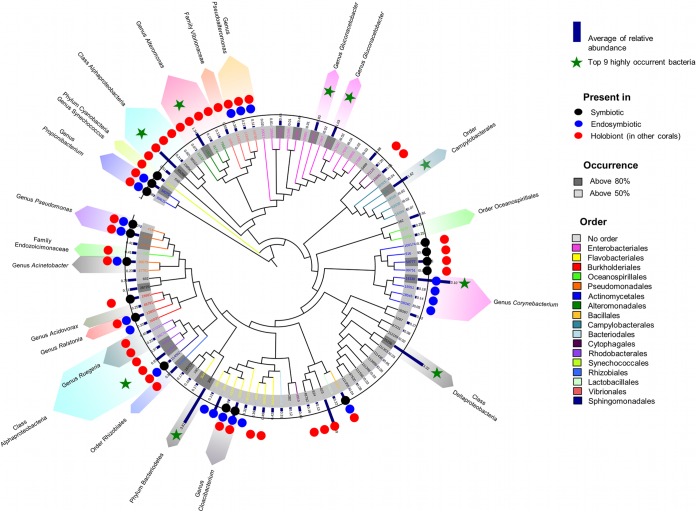
Dendrogram (Tree of Life) of 97 bacteria with high percentages of occurrence (≥50%). Circles represent the microhabitats (symbiotic, endosymbiotic, and holobiont) where OTUs have been reported previously (NCBI, identities ≥97%). Bacterial groups identified as relevant in coral microbial literature are highlighted with color rows. See Table S7 and Table S8 in the supplemental material for complete taxonomic identification and relevant citations.

In the current study, bacterial phylotypes persistently found in over 50% of the samples (designated the core microbiome in *P. speciosa*) predominantly belong to the phyla *Proteobacteria* (61.9%), *Actinobacteria* (10.3%), *Bacteriodetes* (17.5%), *Cyanobacteria* (1%), and *Firmicutes* (2.1%). In the phylum *Proteobacteria*, 53.3% of the core OTUs are class *Gammaproteobacteria*, whereas *Alphaproteobacteria* (16.7%), *Betaproteobacteria* (6.7%), *Deltaproteobacteria* (13.3%), and *Epsilonproteobacteria* (8.3%) have lower percentages of representation (see Tables S7 and S8 in the supplemental material). From nine highly persistent bacterial phylotypes (defined as core due to their presence in ≥90% of all coral colonies) (Fig. 4), four were identified as belonging to genera *Corynebacterium*, *Alteromonas*, and *Gluconacetobacter*, whereas the rest were assigned to higher taxonomic levels. The phylotypes with the highest levels of occurrence, OTUs 306 and 25296, were identified as phylum *Bacteriodetes* and class *Deltaproteobacteria*, respectively (Fig. 4) and are novel in reports on corals (Fig. 3). OTUs 142, 84944, and 65268 were assigned to orders *Campylobacterales* and *Rhodobacteraceae* and class *Alphaproteobacteria*, respectively; the last two were found as part of the holobiont bacteria community in previous studies (31, 32), whereas OTU 142 is also novel in reports on corals.

**FIG 5:**
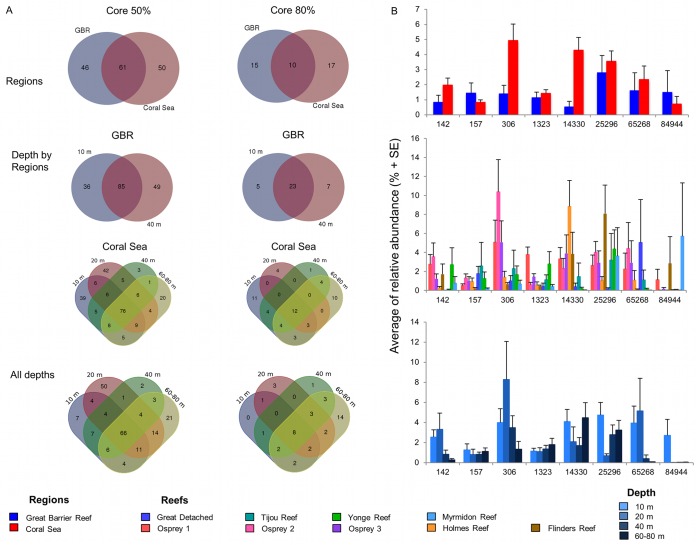
Presence of the eight highly persistent bacteria in coral core microbiome**.** (A) Venn diagrams of occurrence by region, depth by region, and all depths using 50% and 80% as percentages of occurrence to define coral core microbiome. Numbers inside the areas represent the number of OTUs that are part of the core microbiome, and numbers in the intersections represent the number of OTUs in common. (B) Average relative abundances of the eight highly persistent bacteria between regions and reefs and along depth gradients. See Table S7 in the supplemental material for complete taxonomic identification.

Eight of the nine highly persistent and ubiquitous bacteria are present in both reef regions (Coral Sea and GBR) and at all depths (10 to 80 m), but the abundance of each phylotype was found to vary between regions and reefs and along the depth gradient (Fig. 5A and B; see also Table S8 in the supplemental material). Three of the core bacteria have previously been shown to contribute to defense against pathogens and to nutrient intake in other organisms. Members of the genus *Corynebacterium* are able to take up and metabolize urea (33) as a nitrogen source and to synthesize pyrazine (34), a precursor of antibiotic, antitumor, and diuretic substances in humans. Members of the genus *Alteromonas* have been reported as part of coral mucus and skeleton bacterial communities and are able to metabolize dimethylsulfide (DMS) (6), a key organic compound in the cycling of sulfur, and to incorporate and translocate nitrogen into zooxanthellae in coral larvae (35). Moreover, members of the genus *Alteromonas* are able to produce isatin, an antibiotic (36) and antifungal (37) compound in marine organisms. Bacteria of the genus *Gluconacetobacter* are diazotrophic and colonize intracellular spaces and vascular tissues in sugarcane and rice plants (38). As well as contributing to nitrogen fixation, *Gluconacetobacter* bacteria produce plant growth hormones, improving nutrient acquisition (38) and stimulating plant defense response (39). In corals, this genus has been reported in *Montipora* corals as part of a diazotrophic bacterial community (40); however, these particular phylotypes are novel in the literature on corals. The ubiquity of these eight core bacteria in corals across such vast geographic (several degrees of latitude and two distinct regions) and environmental gradients (10 to 80 m depth gradient) suggests a highly stable symbiosis between corals and these bacterial phylotypes. The identification of potential key bacterial symbioses thus enables us to differentiate important bacteria and gives rise to hypotheses about how and when symbioses occur and how critical functional roles are accomplished. Similarly, determining explicitly conserved interactions across individual corals from different regions and depths can allow us to determine potential interactions that aid coral success under vastly different environmental regimes. However, differences in the annotation of core or ubiquitous bacteria between studies are likely, due to several factors, including the type of host species, reef location, depth of sequencing undertaken, degree of host replication, and methodology or criteria used for determining occurrence across samples (7, 19, 21, 29).

### Spatial variability in core microbiomes.

We further identified the potential for spatially explicit core microbiomes in *P. speciosa*: the annotations of core bacteria were analyzed independently for presence in both 50% and 80% of the samples from each region, depth, and depth by region. The GBR and CS corals were found to have different core microbiome communities (Fig. 5); however, there is a group of bacteria that are common and therefore independent of the environmental variation between regions. Similar outcomes were observed for depth gradient per region and at all depths (Fig. 5). For example, the 80% core microbiome for each of the ocean depths (i.e., determined separately between 10 m and 80 m) is constituted principally of the eight highly persistent (core) bacteria, thereby providing evidence for a symbiosis that apparently is able to adjust to different environmental conditions. An additional 14 phylotypes are also evident in 80% of samples from the 60 to 80 m depth range, suggesting that there is habitat partitioning and ecological diversification in core bacterial associations of corals related to reef depth. Niche differentiation and higher genotypic diversity at mesophotic depths have also been observed for coral hosts and their photosymbiotic partners, the *Symbiodinium* dinoflagellates, and this could indicate a bacterial community adaption, or facilitation, to deep environmental conditions (e.g., light, temperature, and nutrient availability) (42–45).

### Three functionally different fractions of the coral holobiont.

Our data support the hypothesis that *P. speciosa* is represented by three functionally distinct bacterial fractions. The symbiotic fraction is represented by a group of ubiquitous core bacteria that are likely to be highly conserved in corals. These eight ubiquitous bacteria are from the classes *Actinobacteria* and *Alpha*-, *Delta*-, *Epsilon*-, and *Gammaproteobacteria* and the phylum *Bacteriodetes*. A high specificity in host-microbe interactions and symbioses has been observed in other natural systems, as is the case in the squid-vibrio symbiosis. During embryogenesis, the bobtail squid, *Euprymna colopes*, develops appendages covered by cilia through which *Vibrio fischeri* colonization occurs. *E. scolopes* squid do not establish symbiosis with any other bacteria, and the cilium appendages are lost once the symbiosis is established, making this one of the most specific bacterial symbioses studied to date (46). The contributions and mechanisms of selection for highly specific, core interactions also need to be investigated in coral, since by doing so, we can have greater insight into the capacity for bacterial symbioses to provide ecological advantages to coral.

We also provide evidence for a functional niche fraction of the coral microbiome. Bacteria that are persistent in specific environmental regimes that are likely to contribute to coral success in particular habitats are characteristic of this functional niche. For example, in *P. speciosa*, this niche fraction is filled by the 14 phylotypes persistently present in the 60 to 80 m depth range, composed of the classes *Actinobacteria*, *Bacilli*, *Flavobacteriia*, *Synechococcophycideae*, and *Alpha*-, *Beta*-, *Delta*-, and *Gammaproteobacteria*. Many similar examples of functional niches have been presented for plants, where bacteria present in the rhizosphere (habitat directly surrounding the root) assist the plant in overcoming abiotic stresses like drought, high and low temperatures, salinity, flooding, heavy metals, organic pollutants, and nutrient deficiency (reviewed in Selvakumar, et al. [47]). Soil and host type, as well as developmental stage, are factors that influence the rhizospheric microbiome (48–50).

*P. speciosa* also hosts numerous bacteria (hundreds of thousands) whose occurrence and abundance are highly variable, and these are likely to be highly responsive to biotic and abiotic processes occurring at diverse spatial scales. This fraction of the bacterial community could be principally inhabiting coral mucus, which is high in nutrients and has fast turnover and rapidly changing abiotic conditions. The surface mucus environment, being the most external coral microhabitat, is exposed to and directly affected by changes in the marine environment, including nutrient fluctuations in the water column, water flow, and sedimentation (20, 25, 51, 52). These micro- and macroscale conditions likely create a fluctuating biotic and abiotic environment that attracts and supports a large diversity of bacteria that are able to colonize microniches, form biofilms, and utilize nutrients.

The occurrence of 8 bacteria within over 100 individual *P. speciosa* colonies collected from 9 geographically distinct coral reefs and at depths of 10 m to 80 m provides substantial evidence for the existence of a coral core microbiome and stable bacterial symbioses in corals. In order to understand the long-term stability of coral bacterial symbiosis, as well as its universality, it is now crucial to test the hypothesis of core microbiome ubiquity across coral species and across temporal scales. The functional role of the core microbiome will be greatly affected by the host’s environment and the conditions in the microhabitat within the host where these bacteria exist; therefore, it is crucial to determine precisely where in the coral host these bacteria reside. Meta-analysis suggests a high likelihood that these bacteria are found in close association with the coral tissues (see Table S8 in the supplemental material). Our results also provide the first evidence of higher diversity in the bacterial communities and of core microbial associations of corals existing in the mesophotic zone of reefs. Furthermore, the high bacterial diversity in corals collected from deeper reefs suggests that there are functional niches in which corals have the capacity to adapt their microbial associations to suit the environmental conditions and utilize available nutrients. In vulnerable ecosystems, such as coral reefs, the evaluation of hosts and their symbioses in time and space is fundamental to understanding how these organisms and the ecosystems they support will be affected by climate change and to what extent they will be able to overcome it.

## MATERIALS AND METHODS

### Experimental design and sample preservation.

Fragments of the plating coral *P. speciosa* (Fig. 1) were collected from reefs of the Great Barrier Reef and the Coral Sea (Fig. 2A). Fragments (*n* = 123) were collected on the Catlin Seaview Survey expeditions during the period from September to December in 2012 and in November of 2013. Corals were sampled during dives using self-contained underwater breathing apparatus (SCUBA) at shallow and intermediate depths (~10 m, ~20 m, and ~40 m), whereas deep coral samples (60 to 80 m) were collected using a remotely operated vehicle (ROV). Here, we utilized a nested hierarchical design considering the following three factors: (i) Depth (fixed factor), with four levels—10 m (±3 m), 20 m (± 2 m), 40 m (±3 m), and 60 to 80 m; (ii) Region (fixed factor) (Fig. 2A), with two levels—Great Barrier Reef (GBR) and Coral Sea (CS); and (iii) Reef (randomly nested in Region) (Fig. 2A), with nine levels—Great Detached, Tijou Reef, Yonge Reef, and Myrmidon Reef in GBR and Osprey 1 (Dutch Towers), Osprey 2 (Halfway Wall), Osprey 3 (Bigeye Ledge), Holmes Reef, and Flinders Reef in CS. Lower mesophotic depths (60 to 80 m) were only sampled in the Coral Sea, whereas the intermediate depth of 20 m was only sampled at Osprey 1 to 3. Four or five coral fragments were collected per depth in each reef.

Coral fragments (~3 cm^2^) were preserved in salt-saturated 20% dimethyl sulfoxide (DMSO)–0.5 M EDTA and stored at −20°C. Sample collection was under permits supplied by the Great Barrier Reef Marine National Park Authority (Townsville, Australia) and Commonwealth Marine Reserves, Department of the Environment (Hobart, Australia).

### DNA extraction and sequencing.

DNA was extracted from approximately 1.4 g (±0.2 g) of each coral fragment using a modified protocol from the MoBio PowerPlant pro DNA isolation kit (catalog no. 13400-50; MoBio, Carlsbad, CA). As described by Sunagawa et al. (17), the modification of the MoBio protocol consisted of digesting samples in proteinase K (final concentration, ≈0.8 mg ml^−1^; Invitrogen) at 65°C for 30 min after homogenization. The purity and quantity of bacterial DNA were determined using a NanoDrop spectrophotometer (Thermo Scientific, Wilmington, DE) and PCRs. Samples were held at −20°C before PCR amplification.

To determine the composition of the bacterial assemblage and the relative abundances of its members, bacterial 16S rRNA gene amplicons were amplified from genomic template primers 515/806 in a single-step, 30-cycle PCR (HotStarTaq plus master mix kit; Qiagen, United States). PCRs were conducted under the following conditions: 94°C for 3 min, followed by 28 cycles of 94°C for 30 s, 53°C for 40 s, and 72°C for 1 min, followed by a final elongation step at 72°C for 5 min. After the amplification, to check the success of amplification and the relative intensities of the bands, amplicon products were checked in 2% agarose gel, and based on molecular weight and DNA concentrations, amplicon products from different samples were pooled in equal proportions. Pooled samples were purified utilizing calibrated Ampure XP beads and sequenced using the Illumina TruSeq DNA library preparation protocol (MR DNA; Shallowater, TX). Sequences have been submitted to the National Center for Biotechnology Information (NCBI) Short Read Archive (SRA) under the project number PRJNA328211.

### Sequence analysis.

Sequence data were analyzed using Quantitative Insights Into Microbial Ecology (QIIME) (53). Bar codes, primers, and short sequences (<200 bp) were removed, and sequences with ambiguous base calls and with homopolymer runs exceeding 8 bp were discarded. The sequences were denoised and chimeras removed. Operational taxonomic units (OTUs) were defined with clustering at 97% similarity. Taxonomy was assigned to OTUs in QIIME using RDP classifier (54) against a curated GreenGenes database (55). Chloroplast and unidentified OTUs were excluded from the OTU table.

### Statistical analysis and core microbiome.

Statistical analysis and data mining were conducted using PRIMER v7 and PERMANOVA+ (56). As our hypothesis was focused on the determination of highly persistent bacteria in corals, bacteria present in less than 5% of the samples (≤6 samples) were excluded from the analyses, as they were considered rare bacteria in the coral-associated assemblage. This filter reduced the quantity of phylotypes from 173,690 to 4,446 OTUs. Normalized relative abundance was obtained using a fourth root transformation and a standardization by sample by total. To analyze the composition of the bacterial assemblage, the matrix of abundances was converted to presence/absence. For both matrices, significant differences in the bacterial assemblages were identified by permutational multivariate analysis of variance (PERMANOVA) using Bray-Curtis distances and explanatory variables as listed above in “Experimental design and sample preservation.” Observed patterns (significant differences at any level) were evaluated with a pairwise comparison. Statistical significance by the *F* test was assessed with 9,999 permutations. To visualize PERMANOVA results, nonmetric dimensional scaling (nMDS) plots using 95% bootstrap regions and averages for the factor Reef were generated from Bray-Curtis similarity matrices of relative abundance and presence/absence data.

The core microbiomes of all the data and for each of the factors considered in the experimental design and their combinations were identified using QIIME. Phylotypes consistently present in >80% of the samples were considered highly persistent bacteria, a conservative representation of the core microbiome, selected based on previous research on core microbiome annotations (19). Phylotypes present in 50 to 79% of the samples were considered persistent bacteria, whereas OTUs not consistently present in at least 50% of the samples were taken as natural variability across colonies.

A dendrogram was constructed using the Interactive Tree of Life software (http://itol.embl.de) (57, 58) from a phylogenetic tree produced in QIIME. Venn diagrams were generated from 50% and 80% core microbiome data and visualized using Venn diagram software (Bioinformatics and Evolutionary Genomics, http://bioinformatics.psb.ugent.be/webtools/Venn/).

### Meta-analysis.

Using the Basic Local Alignment Search Tool (BLAST) algorithm, sequences of the phylotypes that were part of the 50% and 80% core microbiome were searched against the nucleotide database of the National Center for Biotechnology Information (NCBI). Moreover, to determine whether the 50% and 80% core microbiome phylotypes have been reported as part of specific coral microhabitats, the same sequences were compared with the *Acropora granulosa* nucleotide database (19) using a BLAST search. Based on the nucleotide database, sequences with ≥97% identity were classified in four categories and their combinations: (i) not reported in corals, (ii) symbiotic, (iii) endosymbiotic, and (iv) holobiont (in other corals). The category symbiotic represents the bacteria reported in coral tissue, composed of endosymbiotic and episymbiotic tissue regions, and the category endosymbiotic corresponds to coral endodermal cells, excluding skeleton and mucus (19). The category holobiont constitutes bacteria reported in other coral species as part of the whole bacterial assemblage. Sequences annotated as chloroplast were not considered in the analyses.

### Map of sites.

The map of sites (Fig. 2A) was produced with the software QGIS using the Group Layer “GBRMPA features,” data courtesy of the Great Barrier Reef Marine Park Authority (GBRMPA), Copyright Commonwealth of Australia (2007). Bathymetry was obtained from the “Great Barrier Reef and Coral Sea Bathymetry” data set (59), available at http://www.deepreef.org.

## SUPPLEMENTAL MATERIAL

Table S1  Pairwise comparisons from permutational multivariate analysis of variance (PERMANOVA) using Bray-Curtis distances for the interaction Depth × Reef (Region), presence/absence data. Table S1, DOCX file, 0.1 MB

Table S2  Pairwise comparisons from permutational multivariate analysis of variance (PERMANOVA) using Bray-Curtis distances for the factor Reef (Region) in the region Great Barrier Reef, presence/absence data. Table S2, DOCX file, 0.1 MB

Table S3  Pairwise comparisons from permutational multivariate analysis of variance (PERMANOVA) using Bray-Curtis distances for the factor Reef (Region) in the region Coral Sea, presence/absence data. Table S3, DOCX file, 0.1 MB

Table S4  Pairwise comparisons from permutational multivariate analysis of variance (PERMANOVA) using Bray-Curtis distances for the interaction Depth × Reef (Region), abundance data. Table S4, DOCX file, 0.1 MB

Table S5  Pairwise comparisons from permutational multivariate analysis of variance (PERMANOVA) using Bray-Curtis distances for the factor Reef (Region) in the region Great Barrier Reef, abundance data. Table S5, DOCX file, 0.1 MB

Table S6  Pairwise comparisons from permutational multivariate analysis of variance (PERMANOVA) using Bray-Curtis distances for the factor Reef (Region) in the region Coral Sea, abundance data. Table S6, DOCX file, 0.1 MB

Table S7  Taxonomic identifications assigned to operational taxonomic units (OTU) present in the coral core microbiome (bacterial phylotypes persistently found in over 50% of the samples). Table S7, DOCX file, 0.1 MB

Table S8  Associations of bacteria that are part of the 50% coral core microbiome with coral bacteria reported in published literature. Table S8, DOCX file, 0.1 MB
