# Supplementary material for: The Microbial Signature Provides Insight into the Mechanistic Basis of Coral Success across Reef Habitats
Source: mBio. 2016 Jul 26;7(4):e00560-16. doi: 10.1128/mBio.00560-16 (PMC4981706; doi:10.1128/mBio.00560-16)
Supplement: Table S1 — Pairwise comparisons from permutational multivariate analysis of variance (PERMANOVA) using Bray-Curtis distances for the interaction Depth × Reef (Region), presence/absence data. [file mbo004162912st1.docx]

**Table S1.** Pairwise comparisons from permutational multivariate analysis of variance (PERMANOVA) using Bray-Curtis distances for the interaction Depth x Reef(Region), Presence/Absence data.

| Depth (m) | | 10 vs. 20 | | | | 10 vs. 40 | | | | 10 vs. 60-80 | | | | 20 vs. 40 | | | | 20 vs. 60-80 | | | | 40 vs. 60-80 | | | |
| --- | --- | --- | --- | --- | --- | --- | --- | --- | --- | --- | --- | --- | --- | --- | --- | --- | --- | --- | --- | --- | --- | --- | --- | --- | --- |
| Region | Reef | t | P(perm) | U. perms | P(MC) | t | P(perm) | U. perms | P(MC) | t | P(perm) | U. perms | P(MC) | t | P(perm) | U. perms | P(MC) | t | P(perm) | U. perms | P(MC) | t | P(perm) | U. perms | P(MC) |
| Great Barrier Reef | G. Detached | - | | | | 1.1842 | 0.0426 | 126 | 0.2098 | - | | | | - | | | | - | | | | - | | | |
|  | Tijou Reef | - | | | | 1.0691 | 0.1303 | 126 | 0.3461 | - | | | | - | | | | - | | | | - | | | |
|  | Yonge Reef | - | | | | 1.2503 | 0.0189 | 126 | 0.168 | - | | | | - | | | | - | | | | - | | | |
|  | Myrmidon Reef | - | | | | 1.2249 | 0.0515 | 126 | 0.1761 | - | | | | - | | | | - | | | | - | | | |
| Coral Sea | Osprey 1 | 1.2504 | 0.0533 | 126 | 0.175 | 1.085 | 0.1955 | 126 | 0.3351 | 1.4823 | 0.0078 | 126 | 0.0455 | 1.0913 | 0.2852 | 35 | 0.322 | 0.96708 | 0.5403 | 126 | 0.4813 | 1.1361 | 0.1311 | 126 | 0.2709 |
|  | Osprey 2 | 1.1083 | 0.1547 | 126 | 0.2968 | 1.1551 | 0.0708 | 126 | 0.252 | 1.5653 | 0.0224 | 126 | 0.0416 | 1.0479 | 0.2684 | 126 | 0.3763 | 1.5731 | 0.0076 | 126 | 0.0301 | 1.1598 | 0.0723 | 126 | 0.2279 |
|  | Osprey 3 | 1.1499 | 0.0642 | 126 | 0.2405 | 1.2651 | 0.0284 | 126 | 0.1487 | 1.1875 | 0.0548 | 126 | 0.2047 | 1.0792 | 0.182 | 126 | 0.3242 | 1.214 | 0.0217 | 126 | 0.1895 | 1.2964 | 0.016 | 126 | 0.1375 |
|  | Holmes Reef | - | | | | 1.078 | 0.234 | 126 | 0.3348 | 1.2128 | 0.0801 | 126 | 0.1966 | - | | | | - | | | | 0.97571 | 0.51 | 126 | 0.473 |
|  | Flinders Reef | - | | | | 1.4327 | 0.0169 | 126 | 0.0703 | 1.4555 | 0.0086 | 126 | 0.0649 | - | | | | - | | | | 1.0413 | 0.3993 | 35 | 0.3814 |

P(perm): *P*-value based in permutations, U. perms: Unique permutations, P(MC): Monte Carlo *P*- value.
