# Supplementary material for: The Microbial Signature Provides Insight into the Mechanistic Basis of Coral Success across Reef Habitats
Source: mBio. 2016 Jul 26;7(4):e00560-16. doi: 10.1128/mBio.00560-16 (PMC4981706; doi:10.1128/mBio.00560-16)
Supplement: Table S2 — Pairwise comparisons from permutational multivariate analysis of variance (PERMANOVA) using Bray-Curtis distances for the factor Reef (Region) in the region Great Barrier Reef, presence/absence data. [file mbo004162912st2.docx]

**Table S2.** Pairwise comparisons from permutational multivariate analysis of variance (PERMANOVA) using Bray-Curtis distances for the factor Reef(Region) in the Region Great Barrier Reef, Presence/Absence data.

|  | Great Detached | | | | Tijou Reef | | | | Yonge Reef | | | |
| --- | --- | --- | --- | --- | --- | --- | --- | --- | --- | --- | --- | --- |
|  | t | P(perm) | U. perms | P(MC) | t | P(perm) | U. perms | P(MC) | t | P(perm) | U. perms | P(MC) |
| Tijou Reef | 1.1034 | 0.1061 | 9858 | 0.2568 | - | | | | - | | | |
| Yonge Reef | 1.3909 | 0.0003 | 9861 | 0.0294 | 1.1944 | 0.023 | 9847 | 0.1489 | - | | | |
| Myrmidon Reef | 1.4151 | 0.0004 | 9841 | 0.0211 | 1.1555 | 0.0502 | 9840 | 0.1871 | 1.4136 | 0.0011 | 9864 | 0.0294 |

P(perm): *P*-value based in permutations, U. perms: Unique permutations, P(MC): Monte Carlo *P*- value.
