# Supplementary material for: The Microbial Signature Provides Insight into the Mechanistic Basis of Coral Success across Reef Habitats
Source: mBio. 2016 Jul 26;7(4):e00560-16. doi: 10.1128/mBio.00560-16 (PMC4981706; doi:10.1128/mBio.00560-16)
Supplement: Table S3 — Pairwise comparisons from permutational multivariate analysis of variance (PERMANOVA) using Bray-Curtis distances for the factor Reef (Region) in the region Coral Sea, presence/absence data. [file mbo004162912st3.docx]

**Table S3.** Pairwise comparisons from permutational multivariate analysis of variance (PERMANOVA) using Bray-Curtis distances for the factor Reef(Region) in the Region Coral Sea, Presence/Absence data.

|  | Osprey 1 | | | | Osprey 2 | | | | Osprey 3 | | | | Holmes Reef | | | |
| --- | --- | --- | --- | --- | --- | --- | --- | --- | --- | --- | --- | --- | --- | --- | --- | --- |
|  | t | P(perm) | U. perms | P(MC) | t | P(perm) | U. perms | P(MC) | t | P(perm) | U. perms | P(MC) | t | P(perm) | U. perms | P(MC) |
| Osprey 2 | 1.3694 | 0.0029 | 9864 | 0.0209 | - | | | | - | | | | - | | | |
| Osprey 3 | 1.896 | 0.0001 | 9856 | 0.0001 | 1.5906 | 0.0001 | 9872 | 0.0011 | - | | | | - | | | |
| Holmes Reef | 1.4150 | 0.0061 | 9877 | 0.0187 | 1.4906 | 0.0035 | 9897 | 0.0098 | 1.9558 | 0.0001 | 9878 | 0.0002 | - | | | |
| Flinders Reef | 1.2923 | 0.0212 | 9860 | 0.0518 | 1.4789 | 0.0004 | 9867 | 0.0065 | 1.8461 | 0.0001 | 9863 | 0.0003 | 1.2965 | 0.0348 | 9877 | 0.0628 |

P(perm): *P*-value based in permutations, U. perms: Unique permutations, P(MC): Monte Carlo *P*- value.
