# Supplementary material for: The Microbial Signature Provides Insight into the Mechanistic Basis of Coral Success across Reef Habitats
Source: mBio. 2016 Jul 26;7(4):e00560-16. doi: 10.1128/mBio.00560-16 (PMC4981706; doi:10.1128/mBio.00560-16)
Supplement: Table S4 — Pairwise comparisons from permutational multivariate analysis of variance (PERMANOVA) using Bray-Curtis distances for the interaction Depth × Reef (Region), abundance data. [file mbo004162912st4.docx]

**Table S4.** Pairwise comparisons from permutational multivariate analysis of variance (PERMANOVA) using Bray-Curtis distances for the interaction Depth x Reef(Region), Abundance data.

| Depth (m) | | 10 vs. 20 | | | | 10 vs. 40 | | | | 10 vs. 60-80 | | | | 20 vs. 40 | | | | 20 vs. 60-80 | | | | 40 vs. 60-80 | | | |
| --- | --- | --- | --- | --- | --- | --- | --- | --- | --- | --- | --- | --- | --- | --- | --- | --- | --- | --- | --- | --- | --- | --- | --- | --- | --- |
| Region | Reef | t | P(perm) | U. perms | P(MC) | t | P(perm) | U. perms | P(MC) | t | P(perm) | U. perms | P(MC) | t | P(perm) | U. perms | P(MC) | t | P(perm) | U. perms | P(MC) | t | P(perm) | U. perms | P(MC) |
| Great Barrier Reef | G. Detached | - | | | | 1.1293 | 0.0683 | 126 | 0.2709 | - | | | | - | | | | - | | | | - | | | |
|  | Tijou Reef | - | | | | 1.0783 | 0.0963 | 126 | 0.3318 | - | | | | - | | | | - | | | | - | | | |
|  | Yonge Reef | - | | | | 1.2365 | 0.0083 | 126 | 0.1761 | - | | | | - | | | | - | | | | - | | | |
|  | Myrmidon Reef | - | | | | 1.1968 | 0.0764 | 126 | 0.1925 | - | | | | - | | | | - | | | | - | | | |
| Coral Sea | Osprey 1 | 1.2501 | 0.0646 | 126 | 0.1709 | 1.1185 | 0.1542 | 126 | 0.2846 | 1.4149 | 0.0078 | 126 | 0.071 | 1.1363 | 0.2274 | 35 | 0.282 | 0.96912 | 0.4682 | 126 | 0.4739 | 1.135 | 0.1362 | 126 | 0.2699 |
|  | Osprey 2 | 1.0969 | 0.175 | 126 | 0.3208 | 1.1325 | 0.0726 | 126 | 0.2743 | 1.4826 | 0.0239 | 126 | 0.0565 | 1.0415 | 0.2495 | 126 | 0.3845 | 1.524 | 0.0095 | 126 | 0.0405 | 1.14 | 0.0981 | 126 | 0.2542 |
|  | Osprey 3 | 1.137 | 0.0826 | 126 | 0.259 | 1.2781 | 0.0178 | 126 | 0.1328 | 1.1655 | 0.0492 | 126 | 0.2423 | 1.062 | 0.2343 | 126 | 0.3564 | 1.1652 | 0.0232 | 126 | 0.2337 | 1.2752 | 0.0175 | 126 | 0.1474 |
|  | Holmes Reef | - | | | | 1.0607 | 0.2807 | 126 | 0.3435 | 1.1841 | 0.1076 | 126 | 0.2149 | - | | | | - | | | | 0.98751 | 0.4678 | 126 | 0.4453 |
|  | Flinders Reef | - | | | | 1.4106 | 0.0146 | 126 | 0.0772 | 1.4368 | 0.0089 | 126 | 0.0662 | - | | | | - | | | | 1.0159 | 0.3428 | 35 | 0.425 |

P(perm): *P*-value based in permutations, U. perms: Unique permutations, P(MC): Monte Carlo *P*- value.
