# Supplementary material for: The Microbial Signature Provides Insight into the Mechanistic Basis of Coral Success across Reef Habitats
Source: mBio. 2016 Jul 26;7(4):e00560-16. doi: 10.1128/mBio.00560-16 (PMC4981706; doi:10.1128/mBio.00560-16)
Supplement: Table S5 — Pairwise comparisons from permutational multivariate analysis of variance (PERMANOVA) using Bray-Curtis distances for the factor Reef (Region) in the region Great Barrier Reef, abundance data. [file mbo004162912st5.docx]

**Table S5.** Pairwise comparisons from permutational multivariate analysis of variance (PERMANOVA) using Bray-Curtis distances for the factor Reef(Region) in the Region Great Barrier Reef, Abundance data.

|  | Great Detached | | | | Tijou Reef | | | | Yonge Reef | | | |
| --- | --- | --- | --- | --- | --- | --- | --- | --- | --- | --- | --- | --- |
|  | t | P(perm) | U. perms | P(MC) | t | P(perm) | U. perms | P(MC) | t | P(perm) | U. perms | P(MC) |
| Tijou Reef | 1.1142 | 0.0771 | 9840 | 0.2346 | - | | | | - | | | |
| Yonge Reef | 1.3864 | 0.0004 | 9850 | 0.0288 | 1.1888 | 0.0225 | 9849 | 0.1459 | - | | | |
| Myrmidon Reef | 1.3915 | 0.0006 | 9818 | 0.0239 | 1.1913 | 0.0266 | 9830 | 0.14 | 1.3664 | 0.004 | 9852 | 0.0414 |

P(perm): *P*-value based in permutations, U. perms: Unique permutations, P(MC): Monte Carlo *P*- value.
