# Supplementary material for: The Microbial Signature Provides Insight into the Mechanistic Basis of Coral Success across Reef Habitats
Source: mBio. 2016 Jul 26;7(4):e00560-16. doi: 10.1128/mBio.00560-16 (PMC4981706; doi:10.1128/mBio.00560-16)
Supplement: Table S6 — Pairwise comparisons from permutational multivariate analysis of variance (PERMANOVA) using Bray-Curtis distances for the factor Reef (Region) in the region Coral Sea, abundance data. [file mbo004162912st6.docx]

**Table S6.** Pairwise comparisons from permutational multivariate analysis of variance (PERMANOVA) using Bray-Curtis distances for the factor Reef(Region) in the Region Coral Sea, Abundance data.

|  | Osprey 1 | | | | Osprey 2 | | | | Osprey 3 | | | | Holmes Reef | | | |
| --- | --- | --- | --- | --- | --- | --- | --- | --- | --- | --- | --- | --- | --- | --- | --- | --- |
|  | t | P(perm) | U. perms | P(MC) | t | P(perm) | U. perms | P(MC) | t | P(perm) | U. perms | P(MC) | t | P(perm) | U. perms | P(MC) |
| Osprey 2 | 1.3449 | 0.004 | 9872 | 0.0245 | - | | | | - | | | | - | | | |
| Osprey 3 | 1.72 | 0.0001 | 9846 | 0.0001 | 1.4614 | 0.0003 | 9857 | 0.0053 | - | | | | - | | | |
| Holmes Reef | 1.4158 | 0.0043 | 9868 | 0.0161 | 1.4477 | 0.0064 | 9875 | 0.0145 | 1.8041 | 0.0001 | 9867 | 0.0004 | - | | | |
| Flinders Reef | 1.2836 | 0.0217 | 9892 | 0.061 | 1.4191 | 0.0006 | 9869 | 0.0126 | 1.6826 | 0.0001 | 9856 | 0.0003 | 1.2818 | 0.0344 | 9900 | 0.0742 |

P(perm): *P*-value based in permutations, U. perms: Unique permutations, P(MC): Monte Carlo *P*- value.
