# Supplementary material for: The Microbial Signature Provides Insight into the Mechanistic Basis of Coral Success across Reef Habitats
Source: mBio. 2016 Jul 26;7(4):e00560-16. doi: 10.1128/mBio.00560-16 (PMC4981706; doi:10.1128/mBio.00560-16)
Supplement: Table S7 — Taxonomic identifications assigned to operational taxonomic units (OTU) present in the coral core microbiome (bacterial phylotypes persistently found in over 50% of the samples). [file mbo004162912st7.docx]

**Table S7.** Taxonomic identification assigned to Operational Taxonomic Units (OTU) present in the 50% coral core microbiome.

| **OTU** | **Kingdom** | **Phylum** | **Class** | **Order** | **Family** | **Genus** | **Specie** |
| --- | --- | --- | --- | --- | --- | --- | --- |
| **142 **** | Bacteria | Proteobacteria | Epsilonproteobacteria | Campylobacterales |  |  |  |
| **157 **** | Bacteria | Proteobacteria | Gammaproteobacteria | Enterobacteriales | Enterobacteriaceae | *Gluconacetobacter* |  |
| **180** | Bacteria | Bacteroidetes |  |  |  |  |  |
| **262** | Bacteria | Proteobacteria | Gammaproteobacteria |  |  |  |  |
| **282** | Bacteria |  |  |  |  |  |  |
| **289** | Bacteria | Bacteroidetes | Flavobacteriia | Flavobacteriales | Weeksellaceae |  |  |
| **293** | Bacteria | Bacteroidetes | Flavobacteriia | Flavobacteriales | Weeksellaceae |  |  |
| **304** | Bacteria | Bacteroidetes | Flavobacteriia | Flavobacteriales |  |  |  |
| **306 **** | Bacteria | Bacteroidetes |  |  |  |  |  |
| **409** | Bacteria | Bacteroidetes | Flavobacteriia | Flavobacteriales | Flavobacteriaceae | *Nonlabens* | *sediminis* |
| **414** | Bacteria | Proteobacteria | Gammaproteobacteria | Pseudomonadales | Pseudomonadaceae | *Pseudomonas* | *veronii* |
| **548** | Bacteria | Proteobacteria | Alphaproteobacteria |  |  |  |  |
| **623** | Bacteria |  |  |  |  |  |  |
| **692** | Bacteria | Proteobacteria | Gammaproteobacteria |  |  |  |  |
| **727** | Bacteria | Proteobacteria | Gammaproteobacteria | Oceanospirillales | Endozoicimonaceae |  |  |
| **824** | Bacteria |  |  |  |  |  |  |
| **916** | Bacteria | Actinobacteria | Actinobacteria | Actinomycetales | Mycobacteriaceae | *Mycobacterium* |  |
| **1087** | Bacteria |  |  |  |  |  |  |
| **1159** | Bacteria | Bacteroidetes | Flavobacteriia | Flavobacteriales | Weeksellaceae | *Cloacibacterium* |  |
| **1310** | Bacteria | Bacteroidetes | Flavobacteriia | Flavobacteriales | Weeksellaceae | *Cloacibacterium* |  |
| **1323 **** | Bacteria | Proteobacteria | Gammaproteobacteria | Alteromonadales | Alteromonadaceae | *Alteromonas* |  |
| **1450** | Bacteria | Proteobacteria | Gammaproteobacteria | Vibrionales | Vibrionaceae | *Vibrio* | *fortis* |
| **3128** | Bacteria | Proteobacteria | Gammaproteobacteria | Oceanospirillales | Alcanivoracaceae | *Alcanivorax* |  |
| **3174** | Bacteria | Proteobacteria | Gammaproteobacteria | Alteromonadales | Alteromonadaceae | *Alteromonas* |  |
| **3489** | Bacteria | Bacteroidetes |  |  |  |  |  |
| **4919** | Bacteria | Bacteroidetes | Cytophagia | Cytophagales |  |  |  |
| **6312** | Bacteria | Firmicutes | Bacilli | Bacillales | Staphylococcaceae | *Staphylococcus* | *epidermidis* |
| **7956** | Bacteria | Proteobacteria | Gammaproteobacteria | Alteromonadales | Alteromonadaceae | *Alteromonas* |  |
| **11132** | Bacteria | Proteobacteria | Gammaproteobacteria | Alteromonadales | Alteromonadaceae | *Alteromonas* |  |
| **13855** | Bacteria | Proteobacteria | Betaproteobacteria | Burkholderiales | Oxalobacteraceae | *Ralstonia* |  |
| **14049** | Bacteria | Proteobacteria | Gammaproteobacteria | Enterobacteriales | Enterobacteriaceae | *Gluconacetobacter* |  |
| **14183** | Bacteria | Proteobacteria | Gammaproteobacteria | Vibrionales | Pseudoalteromonadaceae | *Pseudoalteromonas* |  |
| **14330 **** | Bacteria | Actinobacteria | Actinobacteria | Actinomycetales | Corynebacteriaceae | *Corynebacterium* |  |
| **14345** | Bacteria | Proteobacteria | Gammaproteobacteria | Vibrionales | Pseudoalteromonadaceae | *Pseudoalteromonas* |  |
| **14379 *** | Bacteria | Proteobacteria | Gammaproteobacteria | Enterobacteriales | Enterobacteriaceae | *Gluconacetobacter* |  |
| **15394** | Bacteria | Proteobacteria | Epsilonproteobacteria | Campylobacterales |  |  |  |
| **15551** | Bacteria | Actinobacteria | Actinobacteria | Actinomycetales | Corynebacteriaceae | *Corynebacterium* |  |
| **15896** | Bacteria | Proteobacteria | Alphaproteobacteria |  |  |  |  |
| **16005** | Bacteria | Proteobacteria | Betaproteobacteria | Burkholderiales | Oxalobacteraceae |  |  |
| **16616** | Bacteria | Bacteroidetes | Bacteroidia | Bacteroidales |  |  |  |
| **16983** | Bacteria | Bacteroidetes | Flavobacteriia | Flavobacteriales | Flavobacteriaceae |  |  |
| **16991** | Bacteria | Proteobacteria | Gammaproteobacteria | Enterobacteriales | Enterobacteriaceae | *Gluconacetobacter* |  |
| **17550** | Bacteria | Proteobacteria | Gammaproteobacteria | Enterobacteriales | Enterobacteriaceae | *Gluconacetobacter* |  |
| **17607** | Bacteria | Proteobacteria | Gammaproteobacteria | Enterobacteriales | Enterobacteriaceae | *Gluconacetobacter* |  |
| **17643** | Bacteria | Proteobacteria | Gammaproteobacteria | Enterobacteriales | Enterobacteriaceae | *Gluconacetobacter* |  |
| **17680** | Bacteria | Proteobacteria | Gammaproteobacteria | Enterobacteriales | Enterobacteriaceae | *Gluconacetobacter* |  |
| **17701** | Bacteria | Proteobacteria | Gammaproteobacteria | Pseudomonadales | Moraxellaceae | *Acinetobacter* | *guillouiae* |
| **18224** | Bacteria | Firmicutes | Bacilli | Lactobacillales | Aerococcaceae | *Alloiococcus* |  |
| **18545** | Bacteria | Actinobacteria | Actinobacteria | Actinomycetales | Corynebacteriaceae | *Corynebacterium* |  |
| **18722** | Bacteria | Bacteroidetes | Flavobacteriia | Flavobacteriales | Weeksellaceae |  |  |
| **18723** | Bacteria | Bacteroidetes |  |  |  |  |  |
| **18991** | Bacteria | Proteobacteria | Betaproteobacteria | Burkholderiales | Burkholderiaceae |  |  |
| **19526** | Bacteria | Proteobacteria | Epsilonproteobacteria | Campylobacterales |  |  |  |
| **19568** | Bacteria | Proteobacteria | Gammaproteobacteria | Enterobacteriales | Enterobacteriaceae | *Gluconacetobacter* |  |
| **19690** | Bacteria | Bacteroidetes | Cytophagia | Cytophagales | Amoebophilaceae |  |  |
| **19786** | Bacteria | Proteobacteria | Gammaproteobacteria | Oceanospirillales | Alcanivoracaceae | *Alcanivorax* |  |
| **19790** | Bacteria | Proteobacteria | Gammaproteobacteria | Vibrionales | Pseudoalteromonadaceae | *Pseudoalteromonas* |  |
| **19806** | Bacteria | Proteobacteria | Gammaproteobacteria | Enterobacteriales | Enterobacteriaceae | *Gluconacetobacter* |  |
| **20017** | Bacteria | Proteobacteria | Gammaproteobacteria | Enterobacteriales | Enterobacteriaceae | *Gluconacetobacter* |  |
| **20801** | Bacteria | Proteobacteria | Gammaproteobacteria | Enterobacteriales | Enterobacteriaceae | *Gluconacetobacter* |  |
| **21048** | Bacteria | Proteobacteria | Gammaproteobacteria | Enterobacteriales | Enterobacteriaceae | *Gluconacetobacter* |  |
| **21122** | Bacteria | Bacteroidetes | Cytophagia | Cytophagales | Amoebophilaceae | *SGUS912* |  |
| **21136** | Bacteria | Proteobacteria | Gammaproteobacteria | Pseudomonadales | Pseudomonadaceae | *Pseudomonas* |  |
| **22721** | Bacteria | Proteobacteria | Gammaproteobacteria | Pseudomonadales | Pseudomonadaceae | *Pseudomonas* |  |
| **25296 **** | Bacteria | Proteobacteria | Deltaproteobacteria |  |  |  |  |
| **26567** | Bacteria |  |  |  |  |  |  |
| **26725** | Bacteria | Proteobacteria | Gammaproteobacteria |  |  |  |  |
| **30079** | Bacteria | Proteobacteria | Gammaproteobacteria | Pseudomonadales | Moraxellaceae | *Acinetobacter* | *rhizosphaerae* |
| **34075** | Bacteria | Actinobacteria | Actinobacteria | Actinomycetales | Propionibacteriaceae | *Propionibacterium* | *acnes* |
| **38184** | Bacteria | Bacteroidetes | Flavobacteriia | Flavobacteriales | Flavobacteriaceae |  |  |
| **48791** | Bacteria | Proteobacteria | Betaproteobacteria | Burkholderiales | Comamonadaceae | *Acidovorax* | *delafieldii* |
| **48792** | Bacteria |  |  |  |  |  |  |
| **50396** | Bacteria | Proteobacteria | Alphaproteobacteria | Rhizobiales | Hyphomicrobiaceae |  |  |
| **58340** | Bacteria | Actinobacteria | Actinobacteria | Actinomycetales | Corynebacteriaceae | *Corynebacterium* |  |
| **59777** | Bacteria | Actinobacteria | Actinobacteria | Actinomycetales | Mycobacteriaceae | *Mycobacterium* |  |
| **65268 **** | Bacteria | Proteobacteria | Alphaproteobacteria |  |  |  |  |
| **82086** | Bacteria | Cyanobacteria | Synechococcophycideae | Synechococcales | Synechococcaceae | *Synechococcus* |  |
| **84944 **** | Bacteria | Proteobacteria | Alphaproteobacteria | Rhodobacterales | Rhodobacteraceae |  |  |
| **87311** | Bacteria | Proteobacteria | Deltaproteobacteria |  |  |  |  |
| **87630** | Bacteria | Proteobacteria | Deltaproteobacteria |  |  |  |  |
| **88352** | Bacteria | Proteobacteria | Alphaproteobacteria | Rhodobacterales | Rhodobacteraceae |  |  |
| **89274** | Bacteria | Proteobacteria | Alphaproteobacteria | Rhodobacterales | Rhodobacteraceae | *Ruegeria* |  |
| **89625** | Bacteria | Actinobacteria | Actinobacteria | Actinomycetales | Propionibacteriaceae | *Propionibacterium* | *acnes* |
| **92525** | Bacteria | Proteobacteria | Epsilonproteobacteria | Campylobacterales |  |  |  |
| **92538** | Bacteria | Proteobacteria | Epsilonproteobacteria | Campylobacterales |  |  |  |
| **95024** | Bacteria | Proteobacteria | Alphaproteobacteria |  |  |  |  |
| **99751** | Bacteria | Actinobacteria | Actinobacteria | Actinomycetales | Mycobacteriaceae | *Mycobacterium* |  |
| **100174** | Bacteria | Actinobacteria | Actinobacteria | Actinomycetales | Mycobacteriaceae | *Mycobacterium* |  |
| **110333** | Bacteria | Proteobacteria | Alphaproteobacteria | Rhodobacterales | Rhodobacteraceae | *Ruegeria* |  |
| **111353** | Bacteria | Proteobacteria | Deltaproteobacteria |  |  |  |  |
| **111355** | Bacteria | Proteobacteria | Deltaproteobacteria |  |  |  |  |
| **111398** | Bacteria | Proteobacteria | Deltaproteobacteria |  |  |  |  |
| **111436** | Bacteria | Proteobacteria |  |  |  |  |  |
| **111556** | Bacteria |  |  |  |  |  |  |
| **112169** | Bacteria | Proteobacteria | Deltaproteobacteria |  |  |  |  |
| **113899** | Bacteria | Proteobacteria | Deltaproteobacteria |  |  |  |  |
| **114062** | Bacteria | Proteobacteria | Alphaproteobacteria | Sphingomonadales | Sphingomonadaceae | *Sphingobium* |  |

* OTU present in the 80% coral core microbiome. ** OTU present in the 80% coral core microbiome and define as highly persistent bacteria (reported in all the reefs and depths).
