# Supplementary material for: The Microbial Signature Provides Insight into the Mechanistic Basis of Coral Success across Reef Habitats
Source: mBio. 2016 Jul 26;7(4):e00560-16. doi: 10.1128/mBio.00560-16 (PMC4981706; doi:10.1128/mBio.00560-16)
Supplement: Table S8 — Associations of bacteria that are part of the 50% coral core microbiome with coral bacteria reported in published literature. [file mbo004162912st8.docx]

**Table S8.** Association of bacteria part of the 50% coral core microbiome with coral bacteria reported in published literature.

| **OTU** | **P. occurrence (%)** | **Avg. rel. abundance** | **Max. value abundance** | **Symbiotic** | **Endosymbiont** | **Holobiont** |
| --- | --- | --- | --- | --- | --- | --- |
| 306 | 99.19 | 3.81 | 48.93 |  |  |  |
| 25296 | 97.56 | 3.32 | 31.74 |  |  |  |
| 1323 | 96.75 | 1.34 | 11.33 |  |  | *A. granulosa* (HT)(1) |
| 14330 | 95.93 | 3.10 | 32.99 |  | *A. granulosa* (HT)(1) |  |
| 65268 | 95.12 | 2.11 | 48.20 |  |  | *M. braziliensis* (MDC)(2) |
| 142 | 93.50 | 1.62 | 21.08 |  |  |  |
| 84944 | 91.87 | 0.97 | 56.08 |  |  | *P. lutea* (HT)(3) |
| 157 | 91.06 | 1.03 | 24.84 |  |  |  |
| 14379 | 90.24 | 0.83 | 18.23 |  |  |  |
| 21136 | 87.80 | 0.74 | 21.99 | *A. granulosa* (HT)(1) | *A. granulosa* (HT)(1) | *A. granulosa* (HT)(1) |
| 59777 | 86.99 | 0.75 | 16.39 | *A. granulosa* (HT)(1) |  | *A. granulosa* (HT)(1) |
| 50396 | 85.37 | 0.82 | 75.02 |  |  | *P. lutea* (HT)(3) |
| 304 | 82.11 | 0.17 | 2.11 |  |  |  |
| 18723 | 82.11 | 0.17 | 1.99 |  |  |  |
| 22721 | 82.11 | 0.41 | 7.33 |  |  |  |
| 26725 | 82.11 | 0.71 | 18.57 |  |  |  |
| 14049 | 81.30 | 0.45 | 6.00 |  |  |  |
| 34075 | 81.30 | 0.45 | 5.92 | *A. granulosa* (HT)(1) | *A. granulosa* (HT)(1) | *A. granulosa* (HT)(1) |
| 16991 | 80.49 | 0.40 | 6.43 |  |  |  |
| 88352 | 80.49 | 0.37 | 22.85 |  |  | *P. lutea* (HT)(3) |
| 7956 | 78.86 | 0.13 | 2.01 |  |  | *A. granulosa* (HT)(1) |
| 82086 | 78.86 | 0.33 | 16.79 | *A. granulosa* (HT)(1) |  | *A. granulosa* (HT)(1) |
| 293 | 78.05 | 1.57 | 18.06 |  | *A. granulosa* (HT)(1) |  |
| 17643 | 78.05 | 0.04 | 0.39 |  |  |  |
| 623 | 77.24 | 2.69 | 81.56 |  |  | *P. compressa* (MHC)(4), *P. lutea* (SCC)(5) |
| 13855 | 77.24 | 0.44 | 9.32 | *A. granulosa* (HT)(1) | *A. granulosa* (HT)(1) | *A. granulosa* (HT)(1) |
| 180 | 76.42 | 0.16 | 1.79 |  |  |  |
| 1450 | 76.42 | 0.50 | 8.67 |  |  | *A. granulosa* (HT)(1), *P. lutea* (DT)(3) |
| 18991 | 75.61 | 0.45 | 4.83 | *A. granulosa* (HT)(1) |  | *A. granulosa* (HT)(1) |
| 1159 | 74.80 | 1.60 | 17.31 | *A. granulosa* (HT)(1) | *A. granulosa* (HT)(1) | *A. granulosa* (HT)(1) |
| 11132 | 74.80 | 0.07 | 0.63 |  |  | *A. granulosa* (HT)(1) |
| 15896 | 74.80 | 0.21 | 4.89 |  |  | *M. braziliensis* (MDC)(2) |
| 6312 | 73.98 | 0.43 | 14.59 | *A. granulosa* (HT)(1) | *A. granulosa* (HT)(1) | *A. granulosa* (HT)(1) |
| 87630 | 73.98 | 0.08 | 0.77 |  |  |  |
| 16983 | 73.17 | 0.49 | 11.05 |  |  | *A. granulosa* (HT)(1) |
| 30079 | 73.17 | 0.44 | 13.12 | *A. granulosa* (HT)(1) | *A. granulosa* (HT)(1) | *A. granulosa* (HT)(1) |
| 727 | 72.36 | 0.41 | 7.32 |  |  | *A. granulosa* (HT)(1), *P. lutea* (HT)(3) |
| 18545 | 71.54 | 0.14 | 1.28 |  | *A. granulosa* (HT)(1) |  |
| 916 | 70.73 | 0.08 | 1.47 | *A. granulosa* (HT)(1) |  | *A. granulosa* (HT)(1) |
| 38184 | 69.92 | 0.42 | 5.51 |  |  |  |
| 262 | 68.29 | 0.91 | 23.61 |  |  |  |
| 113899 | 68.29 | 0.05 | 0.54 |  |  |  |
| 692 | 67.48 | 0.78 | 14.85 |  |  |  |
| 14183 | 67.48 | 0.30 | 7.52 |  | *A. granulosa* (HT)(1) | *A. granulosa* (HT)(1) |
| 58340 | 67.48 | 0.05 | 0.84 |  | *A. granulosa* (HT)(1) |  |
| 87311 | 67.48 | 0.08 | 1.67 |  |  |  |
| 114062 | 67.48 | 0.28 | 4.80 | *A. granulosa* (HT)(1) | *A. granulosa* (HT)(1) | *A. granulosa* (HT)(1) |
| 26567 | 66.67 | 0.62 | 17.35 |  |  |  |
| 548 | 65.85 | 0.51 | 15.15 |  |  | *A. granulosa* (HT)(1), *O. faveolata* (DT)(6); *D. strigosa* (HT)(7) |
| 3128 | 65.04 | 0.29 | 29.02 |  |  |  |
| 3489 | 65.04 | 0.88 | 38.84 |  |  |  |
| 20017 | 64.23 | 0.02 | 0.20 |  |  |  |
| 89625 | 64.23 | 0.08 | 2.11 | *A. granulosa* (HT)(1) | *A. granulosa* (HT)(1) | *A. granulosa* (HT)(1) |
| 48791 | 63.41 | 0.25 | 4.33 |  |  |  |
| 110333 | 63.41 | 0.45 | 36.10 |  |  | *A. granulosa* (HT)(1), *P. lutea* (DT)(3), *P. meandrina* (P)(8) |
| 17680 | 61.79 | 0.03 | 0.41 |  |  |  |
| 99751 | 61.79 | 0.02 | 0.32 | *A. granulosa* (HT)(1) |  | *A. granulosa* (HT)(1) |
| 111436 | 61.79 | 0.05 | 0.62 |  |  |  |
| 14345 | 60.98 | 0.21 | 4.69 |  | *A. granulosa* (HT)(1) | *A. granulosa* (HT)(1), *P. lutea* (HT)(3) |
| 19690 | 60.98 | 0.51 | 22.59 |  |  | *O. faveolata* (HT)(6) |
| 414 | 60.16 | 0.07 | 2.29 | *A. granulosa* (HT)(1) | *A. granulosa* (HT)(1) | *A. granulosa* (HT)(1) |
| 18224 | 60.16 | 0.22 | 3.48 |  |  |  |
| 1087 | 59.35 | 0.27 | 10.59 |  |  |  |
| 19568 | 59.35 | 0.01 | 0.19 |  |  |  |
| 15394 | 57.72 | 0.07 | 0.96 |  |  |  |
| 17701 | 57.72 | 0.23 | 4.08 |  |  |  |
| 95024 | 57.72 | 0.02 | 0.25 |  |  | *M. braziliensis* (MDC)(2) |
| 19790 | 56.91 | 0.11 | 4.66 |  | *A. granulosa* (HT)(1) | *A. granulosa* (HT)(1) |
| 100174 | 56.91 | 0.02 | 0.19 | *A. granulosa* (HT)(1) |  | *A. granulosa* (HT)(1) |
| 111355 | 56.91 | 0.04 | 0.75 |  |  |  |
| 824 | 56.10 | 0.13 | 3.69 |  |  | *P. compressa* (MHC)(4), *P. lutea* (SCC)(5) |
| 16005 | 56.10 | 0.33 | 37.62 |  | *A. granulosa* (HT)(1) |  |
| 18722 | 56.10 | 0.32 | 4.84 |  | *A. granulosa* (HT)(1) |  |
| 19786 | 56.10 | 0.25 | 11.43 |  |  |  |
| 92538 | 56.10 | 0.02 | 0.59 |  |  |  |
| 3174 | 55.28 | 0.16 | 2.13 |  |  | *A. granulosa* (HT)(1), *P. lutea* (DT)(3) |
| 15551 | 55.28 | 0.18 | 1.88 |  | *A. granulosa* (HT)(1) |  |
| 19526 | 55.28 | 0.02 | 0.73 |  |  |  |
| 89274 | 55.28 | 0.22 | 6.60 |  |  | *A. granulosa* (HT)(1), *A. pruinosa** |
| 92525 | 55.28 | 0.04 | 0.55 |  |  |  |
| 111353 | 55.28 | 0.03 | 0.33 |  |  |  |
| 289 | 54.47 | 0.12 | 2.20 |  | *A. granulosa* (HT)(1) |  |
| 111398 | 54.47 | 0.02 | 0.33 |  |  |  |
| 16616 | 53.66 | 0.31 | 20.09 |  |  |  |
| 282 | 52.85 | 0.12 | 2.40 |  |  |  |
| 1310 | 52.85 | 0.10 | 0.96 | *A. granulosa* (HT)(1) | *A. granulosa* (HT)(1) | *A. granulosa* (HT)(1), *P. meandrina* (OB)(8) |
| 17550 | 52.85 | 0.02 | 0.53 |  |  |  |
| 20801 | 52.85 | 0.01 | 0.16 |  |  |  |
| 17607 | 52.03 | 0.02 | 0.46 |  |  |  |
| 21122 | 52.03 | 0.20 | 5.11 |  |  | *A. granulosa* (HT)(1), *O. franksi* (HT)(7) |
| 112169 | 52.03 | 0.02 | 0.42 |  |  |  |
| 409 | 51.22 | 0.39 | 24.57 |  |  | *A. granulosa* (HT)(1) |
| 4919 | 51.22 | 0.49 | 50.51 |  |  |  |
| 21048 | 51.22 | 0.01 | 0.14 |  |  |  |
| 48792 | 51.22 | 0.16 | 4.29 |  |  | *P. compressa* (MHC)(4), *P. lutea* (SCC)(5) |
| 111556 | 51.22 | 0.02 | 0.26 |  |  |  |
| 19806 | 50.41 | 0.02 | 0.23 |  |  |  |

OTU: Operational Taxonomic Units (OTU), P. occurrence: Percentage of occurrence (%), Avg. rel. abundance: Average of relative abundance, Max. value abundance: Maximum value of abundance.

Symbiotic: reported in coral tissue, Endosymbiotic: present in coral endodermals cells, Holobiont: reported as part of the whole bacterial assemblage. HT: healthy tissue, DT: Diseased tissue, MHC: mucus from healthy coral colony, MDC: mucus from diseased coral colony, SCC: Sponge-covered tissue (competition), OB: Oocyte bundies, P: Planulae. *A. granulosa*: *Acropora granulosa*, *M. braziliensis*: *Mussismilia braziliensis*, *P. lutea*: *Porites lutea*, *P. compressa*: *Porites compressa*, *O. faveolata*: *Orbicella faveolata*, *D. strigosa*: *Diploria strigosa*, *P. meandrina*: *Pocillopora meandrina*, *A. pruinosa*: *Acropora pruinosa*, *O. franksi*: *Orbicella franksi*.

* Direct submission: JQ347405.1. Xu,C.Y., Huang,H. and Yang,J.H.

**References**

1. **Ainsworth TD, Krause L, Bridge T, Torda G, Raina JB, Zakrzewski M, Gates RD, Padilla-Gamino JL, Spalding HL, Smith C, Woolsey ES, Bourne DG, Bongaerts P, Hoegh-Guldberg O, Leggat W.** 2015. The coral core microbiome identifies rare bacterial taxa as ubiquitous endosymbionts. ISME J **9:**2261-2274.

2. **Reis AMM, Araujo SD, Moura RL, Francini RB, Pappas G, Coelho AMA, Kruger RH, Thompson FL.** 2009. Bacterial diversity associated with the Brazilian endemic reef coral *Mussismilia braziliensis*. J Appl Microbiol **106:**1378-1387.

3. **Séré MG, Tortosa P, Chabanet P, Turquet J, Quod J-P, Schleyer MH.** 2013. Bacterial communities associated with *Porites* White Patch Syndrome (PWPS) on three Western Indian Ocean (WIO) coral reefs. PLoS ONE **8:**e83746.

4. **Speck MD, Donachie SP.** 2012. Widespread Oceanospirillaceae bacteria in *Porites* spp. J Mar Biol **2012:**7.

5. **Tang SL, Hong MJ, Liao MH, Jane WN, Chiang PW, Chen CB, Chen CA.** 2011. Bacteria associated with an encrusting sponge (*Terpios hoshinota*) and the corals partially covered by the sponge. Environ Microbiol **13:**1179-1191.

6. **Sunagawa S, DeSantis TZ, Piceno YM, Brodie EL, DeSalvo MK, Voolstra CR, Weil E, Andersen GL, Medina M.** 2009. Bacterial diversity and White Plague Disease-associated community changes in the Caribbean coral *Montastraea faveolata*. ISME J **3:**512-521.

7. **Sunagawa S, Woodley CM, Medina M.** 2010. Threatened corals provide underexplored microbial habitats. Plos One **5:**e9554.

8. **Apprill A, Marlow HQ, Martindale MQ, Rappe MS.** 2009. The onset of microbial associations in the coral *Pocillopora meandrina*. ISME J **3:**685-699.
